# Impact of Long-Term RF-EMF on Oxidative Stress and Neuroinflammation in Aging Brains of C57BL/6 Mice

**DOI:** 10.3390/ijms19072103

**Published:** 2018-07-19

**Authors:** Ye Ji Jeong, Yeonghoon Son, Na-Kyung Han, Hyung-Do Choi, Jeong-Ki Pack, Nam Kim, Yun-Sil Lee, Hae-June Lee

**Affiliations:** 1 Division of Radiation Biomedical Research, Korea Institute of Radiological & Medical Sciences, Seoul 01812, Korea; whyj0914@kirams.re.kr (Y.J.J.); sonyh@kribb.re.kr (Y.S.); gmxvz@hanmail.net (N.-K.H.); 2 Primate Resource Center, Korea Research Institute of Bioscience and Biotechnology (KRIBB), Jeonbuk 56216, Korea; 3 Radio Environment & Monitoring Research Group, ETRI, Daejon 34129, Korea; choihd@etri.re.kr; 4 Department of Radio Sciences and Engineering, College of Engineering, Chungnam National University, Daejon 34134, Korea; jkpack@cnu.ac.kr; 5 College of Electrical and Computer Engineering, Chungbuk National University, Cheongju 28644, Korea; namkim@chungbuk.ac.kr; 6 Graduate School of Pharmaceutical Sciences, Ewha Womans University, Seoul 03760, Korea

**Keywords:** RF-EMF, oxidative stress, DNA damage, neuroinflammation, aged brain

## Abstract

The expansion of mobile phone use has raised questions regarding the possible biological effects of radiofrequency electromagnetic field (RF-EMF) exposure on oxidative stress and brain inflammation. Despite accumulative exposure of humans to radiofrequency electromagnetic fields (RF-EMFs) from mobile phones, their long-term effects on oxidative stress and neuroinflammation in the aging brain have not been studied. In the present study, middle-aged C57BL/6 mice (aged 14 months) were exposed to 1950 MHz electromagnetic fields for 8 months (specific absorption rate (SAR) 5 W/kg, 2 h/day, 5 d/week). Compared with those in the young group, levels of protein (3-nitro-tyrosine) and lipid (4-hydroxy-2-nonenal) oxidative damage markers were significantly increased in the brains of aged mice. In addition, levels of markers for DNA damage (8-hydroxy-2′-deoxyguanosine, p53, p21, γH2AX, and Bax), apoptosis (cleaved caspase-3 and cleaved poly(ADP-ribose) polymerase 1 (PARP-1)), astrocyte (GFAP), and microglia (Iba-1) were significantly elevated in the brains of aged mice. However, long-term RF-EMF exposure did not change the levels of oxidative stress, DNA damage, apoptosis, astrocyte, or microglia markers in the aged mouse brains. Moreover, long-term RF-EMF exposure did not alter locomotor activity in aged mice. Therefore, these findings indicate that long-term exposure to RF-EMF did not influence age-induced oxidative stress or neuroinflammation in C57BL/6 mice.

## 1. Introduction

As people are continuously exposed to radiofrequency electromagnetic fields (RF-EMFs) from mobile phone use in everyday life, which has increased over the last 10 years, cumulative exposure is also increasing. In addition, considering the proximity of mobile phones to the head, public concern regarding possible harm to the brain has been raised. RF-EMFs are known to interact with biological systems and may cause oxidative stress under certain circumstances. Many neurological diseases, including Alzheimer’s disease and amyotrophic lateral sclerosis, are now recognized to share atypical inflammatory reactions as a major pathological feature [1]. Neuroinflammation can be both a cause and a consequence of chronic oxidative stress. Since free radicals are essential for brain physiological processes and pathological degeneration, RF-EMFs may contribute to the etiology of neurodegenerative disorders.

Aging, a natural biological process, is manifested by gradual accumulation of oxidized biomolecules and damaged cell organelles, leading to progressive loss of structural and functional integrity and increased risk of mortality [2]. Accumulating evidence indicates that oxidative stress is a major physiological inducer of aging [3]. The brain produces highly reactive free radicals due to its high lipid content and higher oxygen demand and is the organ most affected by aging [4]. In previous studies, increased levels of lipid peroxidation have been found in the inferior temporal cortex of the human brain [5], as well as in the hippocampus and cerebellum of aged rodent brain [6]. Increased levels of 4-hydroxy-2-nonenal (4-HNE) have also been found in Alzheimer’s and Parkinson’s disease [7]; 4-HNE induces peroxidation of linoleic acid and is more stable and can lead to greater damage than can free radicals [8]. Increased oxidation in the brain with age has been demonstrated by measuring levels of protein carbonyl groups in the human cerebral cortex, high levels of which were found in the hippocampus of elderly patients with memory loss. Measuring the 3-nitro-tyrosine (3-NT) protein level is another method of assessing the oxidative modification of proteins. Increased 3-NT levels have been identified in senescent monkey white matter, as well as in the aged brains of other animals [9,10,11]. Furthermore, several studies have found increased levels of 8-hydroxy-2′-deoxyguanosine (8-OHdG) [12,13], including in the postmortem brains of aged subjects [14]. Moreover, brain aging is often associated with chronic and low-grade neurodegeneration and neuroinflammation [15].

Currently, there is a debate regarding the effects of RF-EMFs on DNA damage; some studies have reported harmful effects [16,17,18], while others have reported no significant influence [19,20,21]. In addition, a number of previous studies have suggested that RF-EMFs emitted from mobile phones mediate stress responses through induction of stress proteins, including heat shock proteins (HSPs), which adversely affect cellular homeostasis [22,23]. Neuroinflammation induced by exposure to RF-EMFs has also been reported but remains a matter of debate [24,25,26]. Furthermore, information related to the biological effects of radiofrequency (RF) radiation is still very minimal, and studies related to these effects on DNA damage and neuroinflammation are still controversial. Therefore, it is necessary to investigate whether long-term exposure to RF-EMFs affects oxidative stress and neuroinflammation in the aging brain. The present study was designed to examine the effect of RF-EMF on age-induced expression of markers for oxidative stress (3-NT and 4-HNE), DNA damage (8-OHdG, p53, p27, p21, γH2AX, Bax, and Bcl-2), apoptosis (cleaved caspase-3 and cleaved poly(ADP-ribose) polymerase 1 (PARP-1)), and glial activation (GFAP and Iba-1) in the mouse brain.

## 2. Results

### 2.1. Effect of Long-Term RF Exposure on Age-Induced Oxidative Stress Markers in the Mouse Brain

**Figure 1:**
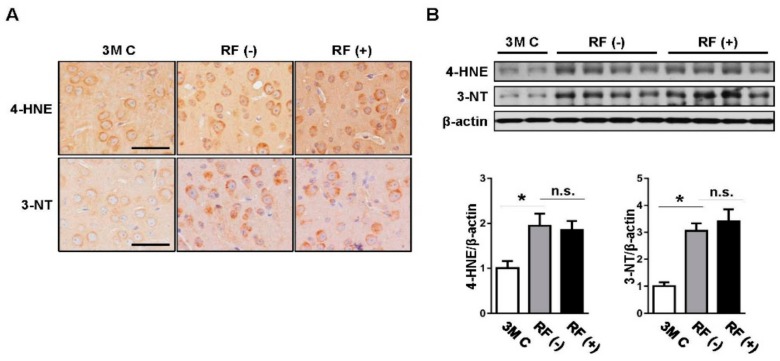
Effects of long-term RF-EMF exposure on induction of oxidative damage in aged mouse brains. (**A**) Representative images of lipid peroxidation (4-HNE, upper panel) and protein nitration (3-NT, lower panel) staining in the young-aged (3M C, *n* = 3), sham-exposed (RF (−), *n* = 6), and RF-exposed (RF (+), *n* = 6) mouse brain. Scale bar: 50 μm. (**B**) Western blot images and subsequent quantification graph of 4-HNE and 3-NT. The values are presented as means ± standard error of the mean (SEM). * *p* < 0.05 versus young-aged (3M C) group. n.s.: no significance.

Oxidative damage was detected by examining the accumulation of 4-HNE and 3-NT as markers of lipid peroxidation and protein nitration, respectively. Immunohistochemical staining illustrated that expression levels were remarkably higher in aged brains than in young (3-month-old) brains (*p* < 0.05), while no difference was observed between the sham-exposed and RF-exposed groups (Figure 1A). Consistent with the immunohistochemistry results, western blot analysis showed that the RF-EMF did not alter the expression of the two oxidative markers compared with their levels in the sham-exposed group (Figure 1B), suggesting that RF-EMF does not alter age-induced oxidative damage in the mouse brain.

### 2.2. Effect of Long-Term RF Exposure on Age-Induced DNA Damage

**Figure 2:**
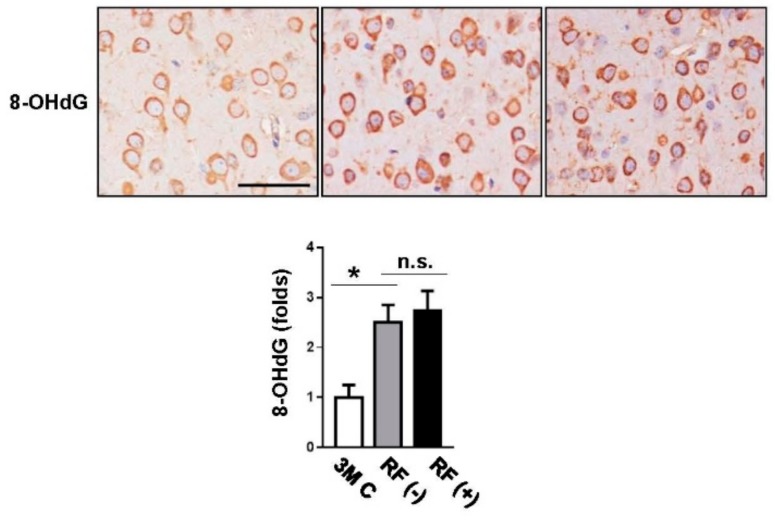
Effects of RF-EMF exposure on the protein expression levels of 8-hydroxy-2′-deoxyguanosine (8-OHdG) as determined by histological analysis. Representative images of DNA damage in the sham-exposed (RF (−), *n* = 6) and 8-month RF-exposed (RF (+), *n* = 6) mouse brain. The expression of 8-OHdG was examined by immunohistochemical staining (**upper panel**) and subsequent quantification (**lower panel**). Both sham-exposed and RF-exposed groups showed increased 8-OHdG expression compared with that in young-aged controls (3M C, *n* = 3). Scale bar: 50 μm. The values are presented as means ± SEM. * *p* < 0.05 versus young-aged (3M C) group. n.s.: no significance.

**Figure 3:**
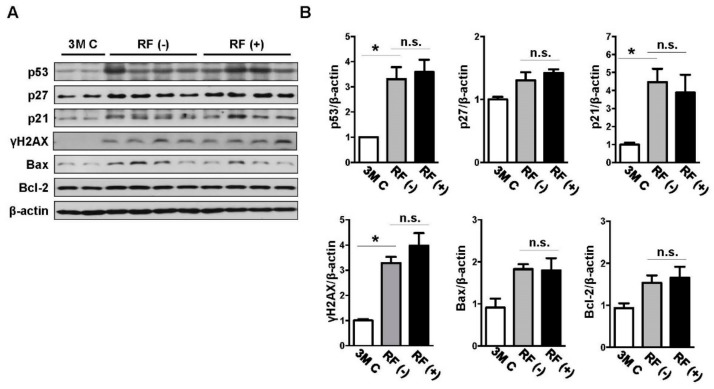
Effects of RF-EMF exposure on the protein expression levels of p53, p27, p21, γH2AX, Bax, and Bcl-2 as determined by western blotting analysis. (**A**) Western blot images of DNA damage-related proteins in the mouse brain following RF-EMF exposure. (**B**) The graphs show the quantification of p53, p27, p21, γH2AX, Bax, and Bcl-2 protein levels in young-aged (3M C), sham-exposed (RF (−)), and RF-exposed (RF (+)) mouse brains based on band intensity. The values are presented as means ± SEM. * *p* < 0.05 versus young-aged (3M C) group. n.s.: no significance.

To assess the effects of RF-EMF exposure on markers of age-induced DNA damage, immunohistochemical analysis of 8-OHdG in the cortex tissue was performed. Its expression showed age-related increases in the mouse brain, but no differences were found between levels in the sham- and RF-exposed groups (Figure 2). In addition, western blotting was performed to examine the protein levels of p53, p27, p21, γH2AX, Bax, and Bcl-2 in the mouse brain following RF-EMF exposure, and β-actin was used as the reference protein (Figure 3). The expression levels of p53, p27, p21, γH2AX, Bax, and Bcl-2 tended to increase in aged brain compared with those in young brain, although the increase in the levels of p27, Bax, and Bcl-2 was not statistically significant. After long-term RF-EMF exposure, there were no differences in the levels of p53, p27, p21, γH2AX, Bax, and Bcl-2 in brain tissue of RF-EMF-exposed mice compared with those in sham-exposed age-matched controls.

### 2.3. Effects of Long-Term RF Exposure on Cleaved Caspase-3 and PARP-1 Expression in the Mouse Brain

**Figure 4:**
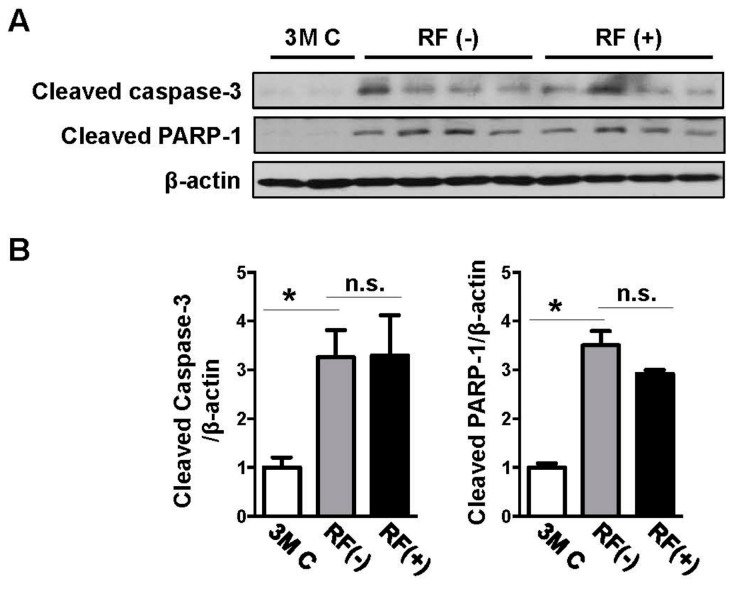
Effects of RF-EMF exposure on the protein expression levels of cleaved caspase-3 and cleaved PARP-1 as determined by western blotting analysis. (**A**) Western blotting images of cleaved caspase-3 and cleaved PARP-1 in the mouse brain following RF-EMF exposure. (**B**) The graphs show the quantification of cleaved caspase-3 and PARP-1 levels in the young-aged (3M C), sham-exposed (RF (−)), and RF-exposed (RF (+)) mouse brain based on band intensity. The values are presented as means ± SEM. * *p* < 0.05 versus young-aged (3M C) group. n.s.: no significance.

Western blotting analysis was performed to examine changes in cleaved caspase-3 and cleaved PARP-1 expression in the mouse brain following long-term RF-EMF exposure. Protein levels increased with aging compared with those in the young-aged group, although the increase in caspase-3 levels was not statistically significant. However, there were no significant differences in cleaved caspase-3 and cleaved PARP-1 expression between the sham- and RF-exposed mice, indicating that long-term RF-EMF exposure did not accelerate age-related apoptotic death in the mouse brain (Figure 4).

### 2.4. Effect of Long-Term RF Exposure on GFAP and Iba-1 in the Mouse Brain

**Figure 5:**
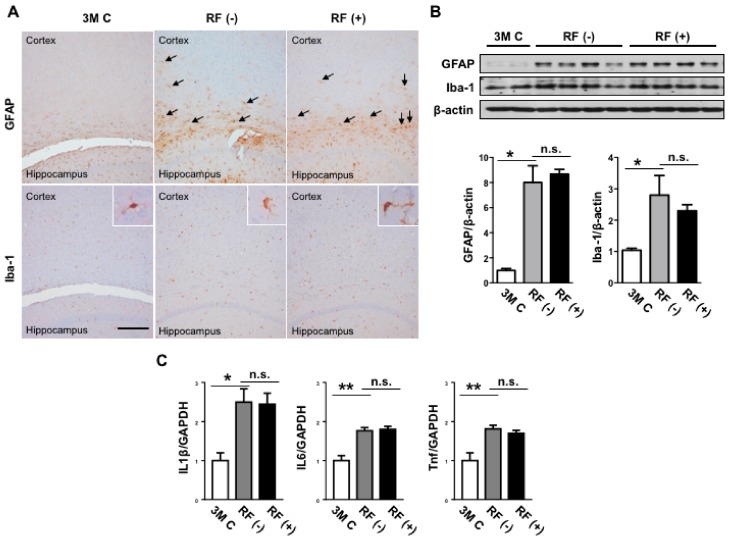
Effects of RF-EMF exposure on the protein expression levels of GFAP and Iba-1 in the aged mouse brain. (**A**) Representative images of astrocytes expressing glial fibrillary acidic protein (GFAP, **upper panel**) and microglia expressing Iba-1 (**lower panel**) in the mouse brain following RF-EMF exposure. Black arrow shows increased expression of GFAP in the cortex area. Inserts show microglia from the cortex. Scale bar: 200 μm. (**B**) Western blot images of GFAP and Iba-1 following RF-EMF exposure. The graphs show the quantification of GFAP and Iba-1 protein levels in the young-aged (3M C), sham-exposed (RF (−)), and RF-exposed (RF (+)) mouse brain based on band intensity. (**C**) Changes in the levels of mRNAs encoding IL-1β, IL-6, TNF in the mouse brain. The values are presented as means ± SEM. * *p* < 0.05 and ** *p* < 0.01 versus young-aged (3M C) group. n.s.: no significance.

To elucidate changes in neuroinflammation in the mouse brain following RF-EMF exposure, the expression levels of the astrocyte marker GFAP and microglia marker Iba-1 were examined by immunohistochemistry. Both GFAP and Iba-1 expression levels increased significantly with age (Figure 5A,B); however, there was no difference between expression levels in the sham- and RF-exposed brains. Consistent with the results of immunohistochemistry, western blotting analysis showed that long-term RF-EMF exposure did not affect GFAP or Iba-1 expression (Figure 5B). Thus, 8 months of RF-EMF exposure did not affect age-related changes in GFAP or Iba-1 expression in the mouse brain.

### 2.5. Effect of Long-Term RF Exposure on Locomotor Activity

**Figure 6:**
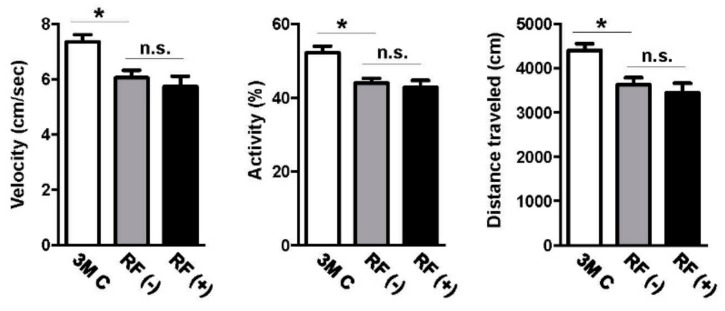
Effects of RF-EMF exposure on general locomotor behavior of aged mice. The young-aged control (3M C, *n* = 3) group showed increased locomotor activity compared with that of aged mice. However, average velocity (**left**), activity (**center**), and total track length (**right**) did not differ between sham-exposed (RF (−), *n* = 12) and RF-exposed (RF (+), *n* = 12) groups. The values are presented as means ± SEM. * *p* < 0.05 versus young-aged (3M C) group. n.s.: no significance.

We explored whether long-term RF-EMF exposure affected basic locomotor activity of the mice by an open field test. The young-aged group of 3-month-old mice showed significantly higher velocity and activity as well as a longer traveled distance than did aged mice (*p* < 0.05). However, no difference between exploratory locomotor activities (measured by average velocity, activity, and total distance traveled) was found in sham- and RF-EMF-exposed mice at the age of 22 months (Figure 6).

## 3. Discussion

Public concern has been raised regarding the possible health risks of RF-EMFs emitted from mobile phones and Wi-Fi devices, since the use of such devices has increased exponentially in daily life. In addition, exposure to RF-EMF has gradually increased in aging people. Aging is associated with elevated oxidative damage to DNA, proteins, and lipids as a result of unbalanced pro-oxidant and antioxidant activities [3]. A previous study showed that oxidative damage associated with aging as well as several neurodegenerative diseases has deleterious effects on brain tissue [27]. Another study reported that RF exposure at 1800 MHz slightly elevated the concentration of malondialdehyde (MDA) in the brain [28], and a 900 MHz RF-EMF altered oxidative stress but was corrected by withdrawal in male rats aged 16–18 weeks [29]. In the present study, to elucidate whether long-term RF-EMF accelerates oxidative damage in the aging mouse brain, we detected accumulation of 4-HNE, a marker of lipid peroxidation, and 3-NT, a marker of oxidative modification of proteins, in the aged mouse brain. We did not observe differences in oxidative stress levels after RF-EMF exposure, indicating that long-term RF-EMF exposure did not influence age-related oxidative damage in the mouse brain. Although several studies reported that RF-EMF exposure augments oxidative stress in the brain [30], we found no such evidence. This discrepancy in the data may be due to differences in the frequency studied (900 or 1800 vs. 1950 MHz), specific absorption rate (SAR, 2 vs. 5 W/kg), exposure method (localized to the brain vs. whole body), and exposure time (up to 60 days vs. up to 8 months). In addition, there were also differences in age (3 vs. 14 months) at the start of RF-EMF exposure. Therefore, further studies examining temporal changes in oxidative stress after long-term exposure of mice of different ages to RF-EMFs are warranted.

A number of studies have shown the possible adverse biological effects of RF-EMFs on DNA, including an increased risk of cancer. A previous in vitro study reported that RF exposure at 1800 MHz induced single-strand DNA breaks in human lens epithelial cells based on an alkaline comet assay [16]. Furthermore, oxidative damage and mitochondrial DNA defects were detected in primary cultured neurons after 1800 MHz RF exposure [17]. Activation of apoptosis is also considered to be involved in possible damage induced by RF-EMF. An in vitro study reported that 1950 MHz signals induced apoptosis in astrocytes with the involvement of Bax and Bcl-2 [31]. In addition, the expression levels of p53 and p21 were changed in the brains of mice after RF exposure [32]. However, in this study, RF-EMF exposure did not seem to affect the expression of markers for DNA damage in the aged mice. Furthermore, no differences were found in the expression of markers for apoptosis. Thus, these data suggest that 8 months of RF-EMF exposure did not affect DNA damage and apoptosis in the brains of mice. Consistent with our findings, a previous in vivo study showed that DNA damage and apoptosis in rat brains were not affected by exposure to RF-EMFs [33,34]. More comprehensive studies on the effects of long-term RF-EMF exposures of varying duration on neurobiological functions are necessary.

It was previously suggested that astrocytes and microglia were responsive to RF-EMFs. Astrocytes and microglia contribute to the optimal functioning of neurons in the healthy brain, and altered function of either cells impacts neuronal function and consequently cognitive function [35]. It is speculated that any changes in glial activation during aging are key components that influence the pathogenesis of neurodegeneration [36]. Loss of astroglial function and astroglial reactivity contributes to the aging of the brain and to neurodegenerative diseases [37]. An increase in the GFAP expression level was observed after acute and chronic RF-EMF exposure [38,39]. A previous in vitro study indicated that microglia and astrocytes were activated by exposure to an 1800 MHz RF-EMF [40]. However, no changes in the levels of GFAP were observed after a single exposure to 1800 MHz signals [24]. GFAP immunostaining in the cortex and hippocampus showed no changes after the heads of both adult and aged rats were exposed to RF-EMF [41]. In addition, a previous study detected no changes in the Iba-1 expression of microglia in mouse brains after acute or chronic exposure to RF-EMFs based on Iba-1 immunostaining [26]. Consistent with the previous studies showing negligible effects on GFAP and Iba-1 expression levels [26,41], the present study found no differences in the protein levels of GFAP and Iba-1. Thus, these data suggest that 8 months of RF-EMF exposure did not affect age-related increases in astrocyte and microglia marker expression in the mouse brain.

Previous studies have reported that RF-EMFs might affect behavioral function. Nevertheless, there are limited data on the long-term effects of RF-EMFs on basic locomotor activity. Increased anxiety and reduced learning behavior were observed when rats were exposed to a 900 MHz signal for 15 days [42]. However, other studies have reported that exposure to 900–2450 MHz signals did not induce spatial or non-spatial memory impairment in rats [43] or mice [44]. Previous studies reported that sub-chronic RF-EMF exposure (during 3 months) did not alter locomotor activity and glial cells (GFAP-positive astrocyte and Iba1-positive microglia) upon exposure of young mice (12 weeks old) to RF-EMF [45]. Further, following 7 months of RF-EMF exposure in young adult mice, no changes were observed in protein oxidative damage in mouse brains, indicating that chronic RF-EMF exposure might not induce detrimental effects on young-adult mice [46]. Additionally, previous reports indicated that RF-EMF did not affect memory or anxiety-related behaviors in either young-adult [39,45,47] or aged animals [41,48]. To examine the effects of RF-EMFs on basic locomotor activity, we performed an open field test. In the present study, RF-EMF exposure did not alter anxiety-related behavior or activity in aged mice. Therefore, the effects of RF-EMFs on neurobiological functions remain controversial and will require further investigations with long-term analyses.

The limitation of this study is the use of only female mice to account for the potential aggression among older male mice. Although we previously reported no deleterious effects of RF-EMF exposure in both male and female rats on serum hormonal levels [49], the estrous states of female mice should be considered because they play a key role in pathological conditions. Previous studies have reported the potential effects of RF-EMFs on the female genital system. Female mice exposed to 20 kHz EMFs may experience extended estrous cycles [50], which is one of the most important factors in reducing the gestation period in female animals. However, adult female rats that exposed to 50 Hz of EMF did not display significant changes in 17-beta-estradiol levels or in the morphology and weight of the uterus and ovaries [51]. Furthermore, a previous study reported that 1439 MHz of time division multiple access (TDMA) exerts no estrogenic effects in rats [52], suggesting that the effect of EMFs on the female genital system, including the estrous cycle, might differ in accordance with the frequency, energy, and species. Therefore, we cannot exclude the possibility that the estrus states of female animals influence the results. Moreover, further studies on the precise interactions between RF-EMF exposure and DNA damage and/or neuroinflammation in either young or aged mice among both female and male animals are required to determine the precise mechanisms underlying the effects of chronic RF-EMF exposure.

In this study, we assessed the effect of RF exposure on the aging brain based on the 2010 RF Research Agenda by the World Health Organization (WHO), which focused on the effects of RF on aging and neurodegenerative diseases [53]. The present study investigated the effects of long-term RF-EMF exposure on behavioral function; locomotor activity; and markers for oxidative stress, DNA damage, and glial activation. We found no significant effects on locomotor activity after exposure to a 1950 MHz RF-EMF for 8 months. In addition, we detected no effect of the RF-EMF on age-related oxidative stress, DNA damage, or glial activation in the mouse brain, which suggested that 8 months of 1950 MHz RF-EMF exposure did not change neurobiological functions in C57BL/6 mice. Although no significant changes in RF-EMF were observed in the aging brain, we first reported that RF did not affect oxidative stress and inflammation in the aging brain, both being important components in the brain aging process. Therefore, our results may form a basis to resolve public health issues raised by WHO.

## 4. Materials and Methods

### 4.1. Animals

Female C57BL/6J mice were obtained from Orient Biotech Co., Ltd. (Gyeonggi-do, Korea). The mice were maintained until 14 months of age and then randomly assigned to two groups: sham exposure (RF (−), *n* = 12) and RF-EMF exposure (RF (+), *n* = 12). RF-EMF exposure was initiated at 14 months of age and terminated at 22 months of age. Three-month-old female mice were used as young-aged controls (3M C, *n* = 5). The animals were housed in a specific pathogen-free facility and were allowed access to a normal diet and autoclaved water ad libitum. All mouse procedures were approved by the Institutional Animal Care and Use Committee of the Korea Institute of Radiological and Medical Sciences (IACUC permit number: KIRAMS2015-0038, 9 June 2015).

### 4.2. RF Exposure System

**Figure 7:**
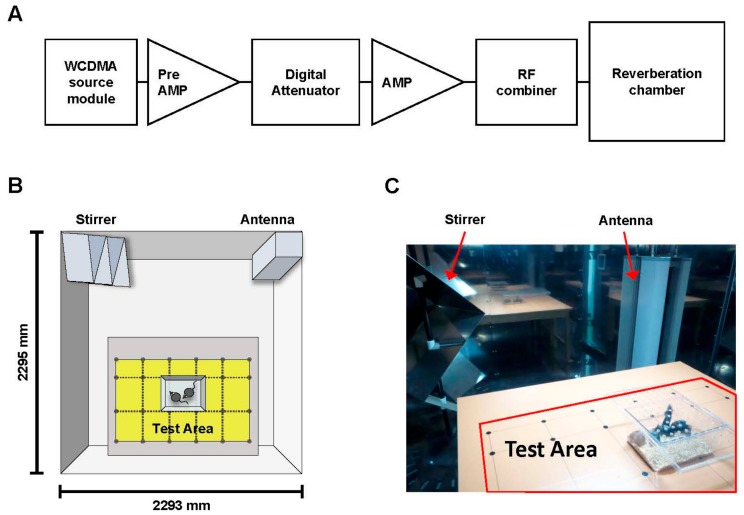
RF-EMF exposure system. (**A**) Schematic flow chart of RF-EMF signal transduction into the reverberation chamber; (**B**) Diagram of RF-EMF exposure test area in the reverberation chamber; (**C**) Photograph of the inner structure of the reverberation chamber.

Whole-body exposure to a 1950 MHz RF-EMF was performed in a reverberation chamber (Figure 7) (ERE-MRC-1.5; ERETEC, Gyeonggi-do, Korea) designed for in vivo experiments. Detailed descriptions of the system, the uniformity of the field dose, and the SAR have been provided previously [54]. The 1950 MHz RF-EMF signal transduction system is depicted in a flow chart in Figure 7A. The RF-EMF was generated using a microprocessor unit chip on which a WCDMA-formatted code controlled a central processing unit as a source module. Subsequently, the signal was amplified using an additional high-power amplifier (PCS60WHPA_CW; Kortcom, Anyang, Gyeonggi-do, Korea) after it was passed through a separate digital attenuator. An 11-bit digital PIN diode attenuator (Model 349; General Microwave, Farmingdale, NY, USA) was used to control the output power level (maximum: 60 W). The transmitting antennae were purchased commercially (patch type, KCAN1900PA; Korea Telecommunication Components, Gyeonggi-do, Korea), and a computer was used to control the exposure level and duration. The external dimensions of the reverberation chamber were 2295 mm × 2293 mm × 1470 mm; the walls were composed of stainless steel with a thickness of 2.3 mm. To measure field uniformity, five cages were placed in the test area, and the field strength was measured for 1 min at 24 points. The distribution of the electric field inside the chamber was determined using a three-axis isotropic probe (HI-6005; ETS-Lindgren, Cedar Park, TX, USA). Whole-body RF-EMF exposure was executed in a test area in which the RF-EMF was transmitted uniformly (Figure 7B,C), and any difference in field distribution was considerably less than 3 dB. The SAR distribution for each caged mouse was calculated using a mouse phantom (Chungnam National University, Daejon, Korea); the simulation featured 40 tissues and a voxel size of 1 mm. The power output was controlled at 52 W to achieve an average whole-body SAR of 5 W/kg. The reverberation chamber was placed in the animal facility, and the ventilation, temperature, and humidity were controlled. The animals were exposed to a 1950 MHz RF-EMF according to the following schedule: SAR 5 W/kg, 2 h/day, 5 d/week for 8 months. The sham-exposed mice were placed inside the chamber for the same period of time without RF-EMF signal. During RF exposure, the air temperature in the test area was maintained at 20 ± 3 °C.

### 4.3. Sample Preparation

The mice were anesthetized by intraperitoneal injection of 30 mg/kg tiletamine-zolazepam (Zoletil; Virbac, Carros, France) and 10 mg/kg xylazine (Rompun; Bayer Korea, Ansan, Korea). Animals underwent cardiac perfusion with phosphate-buffered saline before brain harvest. For histological analysis, the left hemispheres of six mice per group and the whole brains of three mice per group were fixed in 4% paraformaldehyde solution. The other hemispheres of six mice per group and the whole brains of three mice per group were stored at −80 °C for western blotting and quantitative real-time PCR.

### 4.4. Immunohistochemistry

Fixed mouse brains were embedded in paraffin, and embedded tissues were sectioned to 3-μm thickness. The brain from each mouse was sampled at approximately 1.18 mm cranial to the bregma, and a standardized counting area that contained 3 μm thick coronal sections representing the somatosensory cortex region was used. For each mouse, two non-overlapping sections, one from each of the two regions of the cortex approximately 50 μm apart, were analyzed. For antigen retrieval, sections were dewaxed in xylene, rehydrated in gradient alcohol, and boiled in citrate buffer for 30 min. Endogenous peroxidase activity was blocked by incubating the sections in 0.3% H_2_O_2_ in absolute methanol for 15 min at room temperature (RT) for immunoperoxidase labeling, followed by blocking in normal horse serum (S-200; Vector Laboratories, Burlingame, CA, USA). Next, the sections were incubated overnight at 4 °C with each primary antibody: rabbit anti-4-HNE (1:100, ab46545; Abcam, Cambridge, UK), mouse anti-3-NT (1:100, ab7048; Abcam), mouse anti-8-OHdG (1:2500, ab62623; Abcam), goat anti-GFAP (1:500, ab53554; Abcam), and rabbit anti-Iba-1 (1:500, #019-19741; Wako, Osaka, Japan). The following day, sections were washed with 0.1% Triton X-100 in PBS, incubated with each biotinylated secondary antibody for 30 min at RT, and then washed and incubated for 30 min at RT with an avidin–biotin peroxidase complex (PK-6100; Vector Laboratories) prepared according to the manufacturer’s instructions. After the sections were washed, the peroxidase reaction was initiated using a diaminobenzidine substrate (SK-4100; Vector Laboratories) prepared according to the manufacturer’s instructions.

Images of stained sections were captured using a BX-53 microscope equipped with a CCD DP73 digital camera (Olympus, Tokyo, Japan). For 8-OHdG analysis, the images were converted to grayscale, and the threshold was adjusted for every image for background subtraction. The optical density of 8-OHdG in the cortex was identified with ImageJ (NIH, Bethesda, MD, USA). All measurements were performed by the same individual who was blinded to the experimental conditions. The sham exposure group was assigned a mean value of 100, and changes are expressed relative to this value. The relative value per group was averaged and is expressed as mean ± SEM.

### 4.5. Western Blotting

Cortical tissues were treated with tissue lysate buffer (Pro-prep^TM^; Intron Inc., Gyeonggi-do, Korea). Protein concentration was measured by the Bradford assay (Bio-Rad, Hercules, CA, USA). The protein samples were subjected to sodium dodecyl sulfate-polyacrylamide gel electrophoresis (SDS-PAGE) and transferred to a nitrocellulose membrane. Western blot analysis was performed using the following antibodies: rabbit anti-4-HNE (ab46545; Abcam), mouse anti-3-NT (ab7048; Abcam), mouse anti-p53 (#OP09; Calbiochem, San Diego, CA, USA), rabbit anti-p27 (sc-528; Santa Cruz Biotechnology, Dallas, TX, USA), rabbit anti-p21 (#2947; Cell Signaling Technology, Danvers, MA, USA), mouse anti-γH2AX (#05-636; EMD Millipore, Billerica, MA, USA), rabbit anti-Bax (#2772; Cell Signaling Technology), rabbit anti-Bcl-2 (#2876; Cell Signaling Technology), rabbit anti-cleaved caspase-3 (#9661; Cell Signaling Technology), rabbit anti-cleaved PARP-1 (#9542; Cell Signaling Technology), goat anti-GFAP (ab53554; Abcam), rabbit anti-Iba-1 (#016-20001; Wako), and mouse anti-β-actin (Sigma-Aldrich, St. Louis, MO, USA). The next day, the membranes were washed with 0.1% Tween-20 in phosphate-buffered saline (PBS) and incubated with each biotinylated secondary antibody.

### 4.6. Quantitative Real-Time PCR (qRT-PCR)

Total RNA was isolated using TRIzol^®^ Reagent (Molecular Research Center, Cincinnati, OH, USA) and reverse transcribed into cDNA using a ReverTra Ace^®^ qPCR RT kit (TOYOBO, Osaka, Japan), according to the manufacturer’s instructions. qRT-PCR was performed using gene-specific primers with Power SYBR^®^ Green PCR Master Mix (Invitrogen, Carlsbad, CA, USA). The full list of primers used can be found in Table 1. qRT-PCR experiments were performed on the 7300 Real Time PCR System (Applied Biosystems, Foster City, CA, USA).

### 4.7. Open Field Test

The open field test was performed to assay the general locomotive activity of the mice. Mice were placed in the central area of an acrylic box (45 × 45 × 30 cm, width × length × height), and video tracking was started. Mice were allowed to move freely for 10 min in the open field apparatus. The total track length traveled, activity of each mouse, and the time were tracked in the central region of the apparatus and quantified by the tracking program (Viewer3; BIOSERVE GmbH, Mainz, Germany).

### 4.8. Statistical Analysis

Data were analyzed by one-way ANOVA for comparisons between groups, using Graphpad Prism (GraphPad Software, Inc., La Jolla, CA, USA). All data are presented as means ± SEM. Significance was considered at *p*-values less than 0.05.

## Figures and Tables

**Table 1:** Primers used for quantitative real-time PCR (qRT-PCR).

Gene Symbol	Primer Sequence	Product Size (bp)	Source Sequence ID
*IL-1β*	F: 5′-ACCTTTTGACAGTGATGAGAA-3′ R: 5′-GCTGCTGCGAGATTTGA-3′	128	NM_008361.4
*IL-6*	F: 5′-CCTTCCCTACTTCACAAGTC-3′ R: 5′-TTTTCTGCAAGTGCATCATC-3′	187	NM_001314054.1
*TNF*	F: 5′-TGGGTTGTACCTTGTCTACT-3′ R: 5′-TGGTATGAGATAGCAAATCGG-3′	102	NM_013693.3
*GAPDH*	F: 5′-CAAGAAGGTGGTGAAGCAGG-3′ R: 5′-AGGTGGAAGAGTGGGAGTTG-3′	110	NM_008084.3

## References

[1] Mhatre M., Floyd R.A., Hensley K. (2004). Oxidative stress and neuroinflammation in Alzheimer’s disease and amyotrophic lateral sclerosis: Common links and potential therapeutic targets. J. Alzheimer’s Dis..

[2] Lopez-Otin C., Blasco M.A., Partridge L., Serrano M., Kroemer G. (2013). The hallmarks of aging. Cell.

[3] Liu D., Xu Y. (2011). p53, oxidative stress, and aging. Antioxid. Redox Signal..

[4] Gao H.-M., Zhou H., Hong J.-S., Peterson P.K., Toborek M. (2014). Oxidative stress, neuroinflammation, and neurodegeneration. Neuroinflammation and Neurodegeneration.

[5] Dei R., Takeda A., Niwa H., Li M., Nakagomi Y., Watanabe M., Inagaki T., Washimi Y., Yasuda Y., Horie K. (2002). Lipid peroxidation and advanced glycation end products in the brain in normal aging and in Alzheimer’s disease. Acta Neuropathol..

[6] Gemma C., Mesches M.H., Sepesi B., Choo K., Holmes D.B., Bickford P.C. (2002). Diets enriched in foods with high antioxidant activity reverse age-induced decreases in cerebellar beta-adrenergic function and increases in proinflammatory cytokines. J. Neurosci..

[7] Zarkovic K. (2003). 4-hydroxynonenal and neurodegenerative diseases. Mol. Asp. Med..

[8] Ayala A., Munoz M.F., Arguelles S. (2014). Lipid peroxidation: Production, metabolism, and signaling mechanisms of malondialdehyde and 4-hydroxy-2-nonenal. Oxid. Med. Cell. Longev..

[9] Shin C.M., Chung Y.H., Kim M.J., Lee E.Y., Kim E.G., Cha C.I. (2002). Age-related changes in the distribution of nitrotyrosine in the cerebral cortex and hippocampus of rats. Brain Res..

[10] Sloane J.A., Hollander W., Moss M.B., Rosene D.L., Abraham C.R. (1999). Increased microglial activation and protein nitration in white matter of the aging monkey. Neurobiol. Aging.

[11] Tohgi H., Abe T., Yamazaki K., Murata T., Ishizaki E., Isobe C. (1999). Remarkable increase in cerebrospinal fluid 3-nitrotyrosine in patients with sporadic amyotrophic lateral sclerosis. Ann. Neurol..

[12] Agarwal S., Sohal R.S. (1994). Aging and protein oxidative damage. Mech. Ageing Dev..

[13] Richter C., Park J.W., Ames B.N. (1988). Normal oxidative damage to mitochondrial and nuclear DNA is extensive. Proc. Natl. Acad. Sci. USA.

[14] Mecocci P., MacGarvey U., Kaufman A.E., Koontz D., Shoffner J.M., Wallace D.C., Beal M.F. (1993). Oxidative damage to mitochondrial DNA shows marked age-dependent increases in human brain. Ann. Neurol..

[15] Wyss-Coray T. (2016). Ageing, neurodegeneration and brain rejuvenation. Nature.

[16] Yao K., Wu W., Wang K., Ni S., Ye P., Yu Y., Ye J., Sun L. (2008). Electromagnetic noise inhibits radiofrequency radiation-induced DNA damage and reactive oxygen species increase in human lens epithelial cells. Mol. Vis..

[17] Xu S., Zhou Z., Zhang L., Yu Z., Zhang W., Wang Y., Wang X., Li M., Chen Y., Chen C. (2010). Exposure to 1800 MHz radiofrequency radiation induces oxidative damage to mitochondrial DNA in primary cultured neurons. Brain Res..

[18] Kesari K.K., Behari J., Kumar S. (2010). Mutagenic response of 2.45 GHz radiation exposure on rat brain. Int. J. Radiat. Biol..

[19] Stronati L., Testa A., Moquet J., Edwards A., Cordelli E., Villani P., Marino C., Fresegna A.M., Appolloni M., Lloyd D. (2006). 935 MHz cellular phone radiation. An in vitro study of genotoxicity in human lymphocytes. Int. J. Radiat. Biol..

[20] Malyapa R.S., Ahern E.W., Bi C., Straube W.L., LaRegina M., Pickard W.F., Roti Roti J.L. (1998). DNA damage in rat brain cells after in vivo exposure to 2450 MHz electromagnetic radiation and various methods of euthanasia. Radiat. Res..

[21] Heikkinen P., Ernst H., Huuskonen H., Komulainen H., Kumlin T., Maki-Paakkanen J., Puranen L., Juutilainen J. (2006). No effects of radiofrequency radiation on 3-chloro-4-(dichloromethyl)-5-hydroxy-2(5*H*)-furanone-induced tumorigenesis in female Wistar rats. Radiat. Res..

[22] Misa Agustino M.J., Leiro J.M., Jorge Mora M.T., Rodriguez-Gonzalez J.A., Jorge Barreiro F.J., Ares-Pena F.J., Lopez-Martin E. (2012). Electromagnetic fields at 2.45 GHz trigger changes in heat shock proteins 90 and 70 without altering apoptotic activity in rat thyroid gland. Biol. Open.

[23] Yang X.S., He G.L., Hao Y.T., Xiao Y., Chen C.H., Zhang G.B., Yu Z.P. (2012). Exposure to 2.45 GHz electromagnetic fields elicits an HSP-related stress response in rat hippocampus. Brain Res. Bull..

[24] Watilliaux A., Edeline J.M., Leveque P., Jay T.M., Mallat M. (2011). Effect of exposure to 1800 MHz electromagnetic fields on heat shock proteins and glial cells in the brain of developing rats. Neurotox. Res..

[25] Ammari M., Gamez C., Lecomte A., Sakly M., Abdelmelek H., de Seze R. (2010). GFAP expression in the rat brain following sub-chronic exposure to a 900 MHz electromagnetic field signal. Int. J. Radiat. Biol..

[26] Finnie J.W., Cai Z., Manavis J., Helps S., Blumbergs P.C. (2010). Microglial activation as a measure of stress in mouse brains exposed acutely (60 minutes) and long-term (2 years) to mobile telephone radiofrequency fields. Pathology.

[27] Finkel T., Holbrook N.J. (2000). Oxidants, oxidative stress and the biology of ageing. Nature.

[28] Bodera P., Stankiewicz W., Antkowiak B., Paluch M., Kieliszek J., Sobiech J., Niemcewicz M. (2015). Influence of electromagnetic field (1800 MHz) on lipid peroxidation in brain, blood, liver and kidney in rats. Int. J. Occup. Med. Environ. Health.

[29] Ragy M.M. (2015). Effect of exposure and withdrawal of 900-MHz-electromagnetic waves on brain, kidney and liver oxidative stress and some biochemical parameters in male rats. Electromagn. Biol. Med..

[30] Dasdag S., Akdag M.Z. (2016). The link between radiofrequencies emitted from wireless technologies and oxidative stress. J. Chem. Neuroanat..

[31] Liu Y.X., Tai J.L., Li G.Q., Zhang Z.W., Xue J.H., Liu H.S., Zhu H., Cheng J.D., Liu Y.L., Li A.M. (2012). Exposure to 1950-MHz TD-SCDMA electromagnetic fields affects the apoptosis of astrocytes via caspase-3-dependent pathway. PLoS ONE.

[32] Tohidi F.Z., Bahreyni-Toosi M.H., Azimian H., Khademi S., Fardid R., Anani Sarab G. (2015). The gene expression level of p53 and p21 in mouse brain exposed to radiofrequency field. Int. J. Radiat. Res..

[33] Akdag M.Z., Dasdag S., Canturk F., Karabulut D., Caner Y., Adalier N. (2016). Does prolonged radiofrequency radiation emitted from Wi-Fi devices induce DNA damage in various tissues of rats?. J. Chem. Neuroanat..

[34] Yilmaz F., Dasdag S., Akdag M.Z., Kilinc N. (2008). Whole-body exposure of radiation emitted from 900 MHz mobile phones does not seem to affect the levels of anti-apoptotic bcl-2 protein. Electromagn. Biol. Med..

[35] Lynch A.M., Murphy K.J., Deighan B.F., O’Reilly J.A., Gun’ko Y.K., Cowley T.R., Gonzalez-Reyes R.E., Lynch M.A. (2010). The impact of glial activation in the aging brain. Aging Dis..

[36] Luo X.G., Ding J.Q., Chen S.D. (2010). Microglia in the aging brain: Relevance to neurodegeneration. Mol. Neurodegener..

[37] Rodriguez-Arellano J.J., Parpura V., Zorec R., Verkhratsky A. (2016). Astrocytes in physiological aging and Alzheimer’s disease. Neuroscience.

[38] Mausset-Bonnefont A.L., Hirbec H., Bonnefont X., Privat A., Vignon J., de Seze R. (2004). Acute exposure to GSM 900-MHz electromagnetic fields induces glial reactivity and biochemical modifications in the rat brain. Neurobiol. Dis..

[39] Ammari M., Brillaud E., Gamez C., Lecomte A., Sakly M., Abdelmelek H., de Seze R. (2008). Effect of a chronic GSM 900 MHz exposure on glia in the rat brain. Biomed. Pharmacother..

[40] Lu Y., He M., Zhang Y., Xu S., Zhang L., He Y., Chen C., Liu C., Pi H., Yu Z. (2014). Differential pro-inflammatory responses of astrocytes and microglia involve STAT3 activation in response to 1800 MHz radiofrequency fields. PLoS ONE.

[41] Bouji M., Lecomte A., Gamez C., Blazy K., Villegier A.S. (2016). Neurobiological effects of repeated radiofrequency exposures in male senescent rats. Biogerontology.

[42] Saikhedkar N., Bhatnagar M., Jain A., Sukhwal P., Sharma C., Jaiswal N. (2014). Effects of mobile phone radiation (900 MHz radiofrequency) on structure and functions of rat brain. Neurol. Res..

[43] Dubreuil D., Jay T., Edeline J.M. (2003). Head-only exposure to GSM 900-MHz electromagnetic fields does not alter rat’s memory in spatial and non-spatial tasks. Behav. Brain Res..

[44] Yamaguchi H., Tsurita G., Ueno S., Watanabe S., Wake K., Taki M., Nagawa H. (2003). 1439 MHz pulsed TDMA fields affect performance of rats in a T-maze task only when body temperature is elevated. Bioelectromagnetics.

[45] Son Y., Jeong Y.J., Kwon J.H., Choi H.D., Pack J.K., Kim N., Lee Y.S., Lee H.J. (2016). 1950 MHz radiofrequency electromagnetic fields do not aggravate memory deficits in 5xFAD mice. Bioelectromagnetics.

[46] Arendash G.W., Sanchez-Ramos J., Mori T., Mamcarz M., Lin X., Runfeldt M., Wang L., Zhang G., Sava V., Tan J. (2010). Electromagnetic field treatment protects against and reverses cognitive impairment in Alzheimer’s disease mice. J. Alzheimer’s Dis..

[47] Son Y., Jeong Y.J., Kwon J.H., Choi H.D., Pack J.K., Kim N., Lee Y.S., Lee H.J. (2015). The effect of sub-chronic whole-body exposure to a 1950 MHz electromagnetic field on the hippocampus in the mouse brain. JEES.

[48] Klose M., Grote K., Spathmann O., Streckert J., Clemens M., Hansen V.W., Lerchl A. (2014). Effects of early-onset radiofrequency electromagnetic field exposure (GSM 900 MHz) on behavior and memory in rats. Radiat. Res..

[49] Jin Y.B., Choi H.D., Kim B.C., Pack J.K., Kim N., Lee Y.S. (2013). Effects of simultaneous combined exposure to CDMA and WCDMA electromagnetic fields on serum hormone levels in rats. J. Radiat. Res..

[50] Jung K.A., Ahn H.S., Lee Y.S., Gye M.C. (2007). Effect of a 20 kHz sawtooth magnetic field exposure on the estrous cycle in mice. J. Microbiol. Biotechnol..

[51] Aydin M., Cevik A., Kandemir F.M., Yuksel M., Apaydin A.M. (2009). Evaluation of hormonal change, biochemical parameters, and histopathological status of uterus in rats exposed to 50-Hz electromagnetic field. Toxicol. Ind. Health.

[52] Yamashita H., Hata K., Yamaguchi H., Tsurita G., Wake K., Watanabe S., Taki M., Ueno S., Nagawa H. (2010). Short-term exposure to a 1439-MHz TDMA signal exerts no estrogenic effect in rats. Bioelectromagnetics.

[53] Van Deventer E., van Rongen E., Saunders R. (2011). WHO research agenda for radiofrequency fields. Bioelectromagnetics.

[54] Lee H.J., Jin Y.B., Kim T.H., Pack J.K., Kim N., Choi H.D., Lee J.S., Lee Y.S. (2012). The effects of simultaneous combined exposure to CDMA and WCDMA electromagnetic fields on rat testicular function. Bioelectromagnetics.

